# Suppression of Virulence of Toxigenic *Vibrio cholerae* by Anethole through the Cyclic AMP (cAMP)-cAMP Receptor Protein Signaling System

**DOI:** 10.1371/journal.pone.0137529

**Published:** 2015-09-11

**Authors:** M. Shamim Hasan Zahid, Sharda Prasad Awasthi, Masahiro Asakura, Shruti Chatterjee, Atsushi Hinenoya, Shah M. Faruque, Shinji Yamasaki

**Affiliations:** 1 Graduate School of Life and Environmental Sciences, Osaka Prefecture University, Osaka, Japan; 2 Centre for Food and Water Borne Diseases, International Centre for Diarrhoeal Disease Research, Bangladesh, Dhaka, Bangladesh; BRAC, BANGLADESH

## Abstract

Use of natural compounds as antivirulence drugs could be an alternative therapeutic approach to modify the outcome of bacterial infections, particularly in view of growing resistance to available antimicrobials. Here, we show that sub-bactericidal concentration of anethole, a component of sweet fennel seed, could suppress virulence potential in O1 El Tor biotype strains of toxigenic *Vibrio cholerae*, the causative agent of the ongoing 7^th^ cholera pandemic. The expression of cholera toxin (CT) and toxin coregulated pilus (TCP), the major virulence factors of *V*. *cholerae*, is controlled through a regulatory cascade involving activation of ToxT with synergistic coupling interaction of ToxR/ToxS with TcpP/TcpH. We present evidence that anethole inhibits *in vitro* expression of CT and TCP in a *toxT*-dependent but *toxR/toxS*-independent manner and through repression of *tcpP/tcpH*, by using bead-ELISA, western blotting and quantitative real-time RT-PCR assays. The cyclic AMP (cAMP)-cAMP receptor protein (CRP) is a well-studied global signaling system in bacterial pathogens, and this complex is known to suppress expression of *tcpP/tcpH* in *V*. *cholerae*. We find that anethole influences the virulence regulatory cascade by over-expressing *cyaA* and *crp* genes. Moreover, suppression of toxigenic *V*. *cholerae*-mediated fluid accumulation in ligated ileum of rabbit by anethole demonstrates its potentiality as an antivirulence drug candidate against the diseases caused by toxigenic *V*. *cholerae*. Taken altogether, these results revealing a mechanism of virulence inhibition in *V*. *cholerae* by the natural compound anethole, may have relevance in designing antivirulence compounds, particularly against multiple antibiotic resistant bacterial pathogens.

## Introduction

The current status of emergence of multidrug resistant (MDR) pathogenic bacteria has caused loss of the effectiveness of antimicrobial agents against them [1]. Like other bacterial pathogens, multidrug resistance in toxigenic *Vibrio cholerae*, the causative agent of cholera epidemics is a growing concern.

Cholera toxin (CT, encoded by the *ctxAB* genes) is the major virulence factor in toxigenic *V*. *cholerae*, and is mostly responsible for the profuse watery diarrhea leading to severe dehydration [2, 3]. Although over 200 different ‘O’ serogroups of *V*. *cholerae* have already been documented, only O1 (El Tor and classical biotypes) and O139 are responsible for cholera outbreaks [4]. Serogroups other than O1 and O139 are collectively known as non-O1/O139, and associated with occasional cases of diarrhea and extra-intestinal infections [5]. The O1 El Tor biotype of *V*. *cholerae* is responsible for the ongoing 7^th^ cholera pandemic, and this biotype replaced the classical biotype strain which caused the 6^th^ cholera pandemic. Recently emerged *V*. *cholerae* O1 El Tor variant strains (possess some attributes of classical biotype including *ctxB* gene allele) produce more CT and cause more severe symptoms of diarrhea than prototype El Tor [6, 7].

Along with CT, by using another virulence factor toxin-coregulated pilus (TCP, encoded by the *tcp* gene cluster) *V*. *cholerae* causes diarrheal diseases to human host. Although the virulence regulon in toxigenic *V*. *cholerae* was recognized as the ToxR regulon, ToxT is the direct transcriptional activator of the genes encoding CT and TCP. Indeed, activation of *toxT* occurs via synergistic coupling of two membrane-localized heterodimers ToxR/ToxS and TcpP/TcpH [8–10]. Interestingly, over production of TcpP overcomes the requirement for ToxR in activating *toxT*, but the reverse is not true [11]. This suggests that TcpP is more directly responsible for transcriptional activation of *toxT* and that ToxR plays an indirect role. On the other hand, TcpH protects the periplasmic domain of TcpP from proteolytic cleavage, and thus maintain the cellular level of TcpP [12].

Due to declining performance of traditional antibiotics, use of antivirulence drugs could be a novel therapeutic approach to combat diseases caused by toxigenic *V*. *cholerae*. As CT is the major virulence factor in toxigenic *V*. *cholerae*, much attention has been paid to search suitable antivirulence drug candidates against CT. Previous studies demonstrated that bile repressed *ctxA* and *tcpA* transcriptions in a ToxT-independent manner [13], but a synthetic compound virstatin inhibited CT production in a ToxT-dependent manner in *V*. *cholerae* [14]. In another recent study, synthetic compound toxtazin B has been found to affect ToxT by inhibiting *tcpP* transcription, but mechanisms behind *tcpP* inhibition is still obscure [15]. However, there is still very limited information regarding the effects of bioactive compounds from natural sources on the virulence gene regulation in *V*. *cholerae*. In our previous study, we have shown that sub-bactericidal concentration of extracts of some spices, such as red chili, sweet fennel and star anise seed can effectively inhibit CT production in *V*. *cholerae* [16]. Recently, we have also reported that capsaicin, a well-studied component of red chili, drastically suppressed *in vitro* CT production in *V*. *cholerae* in a *toxT*-dependent manner by upregulating *hns* transcription [17], but failed to show such activity *in vivo*.

As potential inhibition of virulence gene expression was observed in toxigenic *V*. *cholerae* by sub-bactericidal concentration of extracts of sweet fennel and star anise seeds, it would be very useful if we could identify the active compounds exerting such effects. First, we targeted trans-anethole (1-methoxy 4-propenyl benzene), which accounts for 80–90% of the essential oil derived from sweet fennel and star anise seeds [18]. In a recent study, we have reported that although ≥ 200 μg/ml of anethole (trans-anethole) is bactericidal, ≤ 100 μg/ml did not show any detectable effect on the growth of toxigenic *V*. *cholerae* strains [19]. In this study, we have evaluated anethole (sub-bactericidal concentration) as a potential inhibitor of virulence factors production in *V*. *cholerae* both *in vitro* and *in vivo*. Furthermore, the possible molecular mechanisms behind anethole-mediated virulence gene inhibition in *V*. *cholerae* were also investigated.

## Materials and Methods

### Bacterial strains, plasmids and culture conditions

A description of different toxigenic *V*. *cholerae* strains, the relevant characteristics of specific gene mutant strains and properties of plasmids used in this study are listed in Table 1. AKI-medium [0.5% NaCl, 0.4% Yeast extract, 1.5% Bactopeptone and 0.3% NaHCO_3_ (pH 7.4)] at 37°C for O1 El Tor/O139 strains [20] and Luria-Bertini (LB) broth [(pH 6.6), Becton, Dickinson and Company, Franklin lakes, NJ] at 30°C for O1 classical strains were used for optimum growth, unless otherwise stated. Among *V*. *cholerae* strains, a representative O1 El Tor variant strain (CRC41) which has been analyzed and characterized in our previous study [17], also used here for studies in details. *Escherichia coli* DH5αλpir and SM10λpir were used for cloning and conjugation study, respectively. Antimicrobials were used at the following concentrations: ampicillin, 100 μg/ml; kanamycin, 30 μg/ml; nalidixic acid, 30 μg/ml.

**Table 1:** Bacterial strains and plasmids used in this study.

Strains or plasmids	Relevant characteristics^a^	Source or reference
**Serial and identity of wild-type *V*. *cholerae* strains**		
**1.** NICED-10	O1 El Tor, *ctxB* genotype: El Tor	India, 1970
**2.** NICED-3	O1 El Tor, *ctxB* genotype: El Tor	India, 1980
**3.** P130	O1 El Tor, *ctxB* genotype: El Tor	Peru, 1991
**4.** VC190	O1 El Tor, *ctxB* genotype: El Tor	India, 1993
**5.** AI-091	O1 El Tor variant, *ctxB* genotype: Classical	Bangladesh, 1993
**6.** CO533	O1 El Tor variant, *ctxB* genotype: Classical	India, 1994
**7.** CRC27	O1 El Tor variant, *ctxB* genotype: Classical	India, 2000
**8.** CRC41	O1 El Tor variant, *ctxB* genotype: Classical	India, 2000
**9.** CRC87	O1 El Tor variant, *ctxB* genotype: Classical	India, 2000
**10.** B33	O1 El Tor variant, *ctxB* genotype: Classical	Mozambique, 2004
**11.** SG24	O139, *ctxB* genotype: El Tor	India, 1992
**12.** CRC142	O139, *ctxB* genotype: Classical	India, 2000
**13.** 569B	O1 classical, *ctxB* genotype: Classical	India, 1948
**14.** O395	O1 classical, *ctxB* genotype: Classical	India, 1964
**Mutant *V*. *cholerae* strains**		
Δ*cyaA*-CRC41	Derivative of El Tor variant strain CRC41 carrying deletion of *cyaA* (VC0122)	This study
	gene encoding adenylate cyclase	
Δ*crp*-CRC41	Derivative of El Tor variant strain CRC41 carrying deletion of *crp* (VC2614)	This study
	gene encoding cAMP receptor protein	
***E*.*coli* strains**		
DH5αλpir	supE44 DlacU169 (/80 lacZDM15) hsdR17 recA1 endA1 gyrA96 thi-1	[27]
	*relA1 (*λ pirR6K)	
SM10λpir	*thi-1*, *thr*, *leu*, *tonA*, *lacY*, *supE*, *recA*::RP4-2-Tc::Mu, Km^r^, (λ pirR6K)	[27]
**Plasmids**		
pWM91	*ori*R6K plasmid vector used for *sacB*-mediated allelic exchange, Amp^r^	[26]
pΔ*cyaA*	pWM91::ΔVC0122, Amp^r^	This study
pΔ*crp*	pWM91::ΔVC2614, Ampr	This study
pCyaA	pBR322 carrying *cyaA* gene including putative promoter region, Amp^r^, Tet^r^	This study
pCRP	pBR322 carrying *crp* gene including putative promoter region, Amp^r^, Tet^r^	This study
pTcpA	pET-28a(+) carrying entire *tcpA* gene, Km^r^	This study
pTcpPH	pET-28a(+) carrying entire *tcpPH* gene, Km^r^	This study

^a^ *ctxB* genotyping of the wild-type *V*. *cholerae* strains was determined as described in our previous study [17].

### Quantification of CT production by bead-ELISA

Based upon the biotype and serogroup, a single colony of *V*. *cholerae* was inoculated either in AKI medium at 37°C or in LB broth at 30°C. After 12 h incubation, optical density (OD) at 600 nm (OD_600_) was adjusted to 1.0. Subsequently, cultures were 100-fold diluted with fresh AKI medium and incubated either in the presence or absence of trans-anethole (Nacalai Tesque, Kyoto, Japan; purity 98%), according to Iwanaga et al. [20], with slight modifications. Briefly, cultures were kept under stationary condition for an initial 4 h and then shifted to a shaking condition at 180 rpm for another 4 h at 37°C, unless otherwise mentioned. Appropriate dilutions of the cell-free supernatant (CFS) of the samples were made with phosphate-buffered saline (PBS, pH 7.0). Dilutions of purified CT of known concentrations were used to estimate the amount of CT in CFS by a bead-ELISA as described previously [21]. Before CFS preparation, bacterial viability from each culture was tested by spreading the PBS-diluted culture on LB-agar. As anethole (trans-anethole) was dissolved and diluted in 99.9% methanol, methanol (≤ 1%) alone was also added in a control assay to determine its effect on bacterial growth and CT production.

### DNA manipulations

As the genomic sequence of the strain CRC41 (O1 El Tor variant) is currently unknown, we used the relatively close published sequence of *V*. *cholerae* O1 El Tor strain N16961 (accession no. AE003852) to design primers for targeted regions of desired genes. Then, by using those primers amplification of targeted genes were carried out from CRC41. The amplified DNA fragments from CRC41 genome were then sequence-verified to see whether there is any mismatch nucleotide present in the sequenced regions compared to those of N16961. Finally, all of the primers and TaqMan probes used in this study were designed based on the CRC41 sequence, and are represented in Table 2. Restriction and DNA modification enzymes were purchased from TaKaRa Bio Inc. (Shiga, Japan). All the PCR amplicons and cloned products from CRC41 were sequence-verified by using ABI PRISM 3100-Avant genetic analyzer (Applied Biosystems Inc., Foster city, CA). The nucleotide sequences were aligned and analyzed by using a Laser-gene DNASTAR (Madison, WI) software package.

**Table 2:** Sequences of oligonucleotides used in this study.

**Primer** ^**a**^ **/probe** ^**a**^ ^,^ ^**b**^	**Sequence (5'-3')** ^**c**^
**For qRT-PCR**	
*ctxA*-rt-F	GGA GGG AAG AGC CGT GGA T
*ctxA*-rt-P	CAT CAT GCA CCG CCG GGT TG
*ctxA*-rt-R	CAT CGA TGA TCT TGG AGC ATT C
*tcpA*-rt-F	GGG ATA TGT TTC CAT TTA TCA ACG T
*tcpA*-rt-P	TGC TTT CGC TGC TGT CGC TGA TCT T
*tcpA*-rt-R	GCG ACA CTC GTT TCG AAA TCA
*toxT*-rt-F	TGA TGA TCT TGA TGC TAT GGA GAA A
*toxT*-rt-P	TAC GCG TAA TTG GCG TTG GGC AG
*toxT*-rt-R	TCA TCC GAT TCG TTC TTA ATT CAC
*toxR*-rt-F	GCT TTC GCG AGC CAT CTC T
*toxR*-rt-P	CTT CAA CCG TTT CCA CTC GGG CG
*toxR*-rt-R	CGA AAC GCG GTT ACC AAT TG
*toxS*-rt-F	TGC CAT TAG GCA GAT ATT TCA CA
*toxS*-rt-P	TGA CGT CTA CCC GAC TGA GTG GCC C
*toxS*-rt-R	GCA ACC GCC CGG CTA T
*tcpP*-rt-F	TGG TAC ACC AAG CAT AAT ACA GAC TAA G
*tcpP*-rt-P	TAC TCT GTG AAT ATC ATC CTG CCC CCT GTC
*tcpP*-rt-R	AGG CCA AAG TGC TTT AAT TAT TTG A
*tcpH*-rt-F	GCC GTG ATT ACA ATG TGT TGA GTA T
*tcpH*-rt-P	TCA ACT CGG CAA AGG TTG TTT TCT CGC
*tcpH*-rt-R	TCA GCC GTT AGC AGC TTG TAA G
*hns*-rt-F	TCG ACC TCG AAG CGC TTA TT
*hns*-rt-P	CTG CGC TAT CAG GCG AAA CTA AAA CGA AA
*hns*-rt-R	GGT GCA CGT TTG CCT TTT G
*cyaA*-rt-F	CAC ACT GCT CAA CCC ACA AAT T
*cyaA*-rt-P	CCC CAG ACC TGC ATG AGC CCG
*cyaA*-rt-R	CCA GCA CAA ACC TCA ATA AAA CTT AA
*crp*-rt-F	GAT GCG CCT TTC AGG TCA A
*crp*-rt-P	TCG TCG TCT GCA AGT GAC CAG CCA
*crp*-rt-R	CGC AAG GTC GCC AAC TTT
*hapR*-rt-F	GCG CAA TCT CGG CAA TAT CT
*hapR*-rt-P	CAC CAC GAC CAA TGC CGC GTT TA
*hapR*-rt-R	AAA TCG CGT TGG AAG TGT TTG
*aphA*-rt-F	GCA GCA ACG TTT AGA GCG TTT
*aphA*-rt-P	CGT CGT AAT TTA CTG GTT CGC CAA GCA
*aphA*-rt-R	TCG TCC GCC CAT TGA ATC
*aphB*-rt-F	GCA TGA GCG TAA TGC CTA AAC C
*aphB*-rt-P	TCT GAA CAT GCG CTG CGA ACA ACA
*aphB*-rt-R	TTC AAG CCA GCG CAC TGA
*rseP*-rt-F	CGG GAA TCG CAC CAA AAG
*rseP*-rt-P	CGC AGA ATG GCC GCA AAA CTA TCG
*rseP*-rt-R	CGA CTC AAA TAC ACC AAA TTG CA
*degS*-rt-F	GCT ACC GGA CGT TCA TCC A
*degS*-rt-P	CGC TGA TGG TCG CCA AGC CTT TAT T
*degS*-rt-R	CAT TGA TTG CGG CAT CAG TTT
**Primer** ^**a**^ **/probe** ^**a**^ ^,^ ^**b**^	**Sequence (5'-3')** ^**c**^
*recA*-rt-F	CAA TTT GGT AAA GGC TCC ATC AT
*recA*-rt-P	CTT AGG CGA CAA CCG CGC
*recA*-rt-R	CCG GTC GAA ATG GTT TCT ACA
**For mutant construction**	
*cyaA*-FO	GACCGGAACATCTTTCATTG
*cyaA*-5O	GATCGGGATCCATAAGCCTTTACCGCCCACT
*cyaA*-5I	AGAGACGACGCGTGCCAGTCCTGCAAGTTTGCTTCCCTG
*cyaA*-3O	GATCGGCGGCCGCCGAGAGCCGACAACAAAAAC
*cyaA*-3I	GACTGGCACGCGTCGTCTCTAGCTCAAGCCCAGTTTTTG
*cyaA*-RO	TATACAGTGGCCCAGTTTGC
*crp-*FO	ATTCCATCGTCCGTTCAATG
*crp*-5O	GATCGCTCGAGATTCCAACGCTGGATGAGAG
*crp*-5I	AGAGACGACGCGTGCCAGTCAGTGTTGGATCGGTTTGAGG
*crp*-3O	GATCGGGATCCTGCAAGCGATTGTTGAAAAG
*crp*-3I	GACTGGCACGCGTCGTCTCTCGGTTCGTGCTTTCAAAGAT
*crp*-RO	CATTTTGAACATCCCGATCC
**For recombinant protein expression**	
TcpA-pr-F	GATCGGGATCCATGCAATTATTAAAACAGC
TcpA-pr-R	GATCGCTCGAGTTAACTGTTACCAAAAGC
TcpPH-pr-F	GATCGGGATCCATGGGGTATGTCCGCGTG
TcpPH-pr-R	GATCGCTCGAGCTAAAAATCGCTTTGAC
**For complementation of protein in mutant strain**	
CyaA-PBR-comp F	GATCGAAGCTTGTGATGTGCTTCCAAGAGC
CyaA-PBR-comp R	GATCGGGATCCTTAGGCATTGACCACTTG
CRP-PBR-comp F	GATCGAAGCTTGCAAATTGGACTACTGACACGA
CRP-PBR-comp R	GATCGGGATCC TTAGCGAGTGCCGTAAACC

^a^All the primers and probes were designed using PRIMER EXPRESS software version 3.0 (Applied Biosystems Inc.).

^b^FAM was used as a 5'-reporter dye and TAMRA as a 3'-quencher dye to design each TaqMan probe.

^c^Underlines below the nucleotide sequences indicate restriction enzyme cleavage sites. F, forward primer; P, probe; R, reverse primer; O, outer primer; I, inner primer.

### Western blot analysis of TcpA and TcpP

To express recombinant TcpA and TcpP, plasmids pTcpA and pTcpPH were constructed, respectively in a His_6_-T7-thrombin digestion site tagged vector pET-28a(+) (Novagen, Madison, WI). The entire *tcpA* (VC0828) and *tcpPH* (VC0826-0827) genes were cloned into the compatible sites of pET-28a(+) vector, and transformed to an *E*. *coli* host. As His_6_-T7-thrombin digestion site is physically linked and coordinately expressed, the expected size of each of the expressed recombinant protein will be ~5-kD higher than their original size. Purified recombinant TcpA and TcpP were used as a positive control to detect TcpA and TcpP expression, respectively in *V*. *cholerae* by western blotting. The nucleotide sequences used for gene cloning are presented in Table 2.

To analyze the effect of anethole on TcpA and TcpP expression, whole-cell lysates were prepared as described previously [22] with some modifications. Briefly, OD_600_ of *V*. *cholerae* cells grown under desired conditions were adjusted to 5.0 in 100 mM Tris-HCl buffer (pH 8.0) containing 100 mM sucrose, and 0.2 mM EDTA. Samples were quickly frozen in liquid nitrogen, thawed in cold water and subjected to DNase I treatment [amplification grade (5 μl of 1 U/μl), Invitrogen, Carlsbad, CA] for 15 min at room temperature. Samples were then treated with protease inhibitor cocktail (Sigma-Aldrich Corporation, St. Louis, MO) and whole-cell lystaes were prepared by ultrasonic treatment (Astrason W-385 ultrasonic processor) with 5 to 7 pulses of 30 sec each. Proteins from equal volume of cell extracts of all samples were separated by SDS-PAGE using 15% (wt/vol) polyacrylamide gels, transferred to a polyvinylidene difluoride (PVDF) membrane in a Trans-blot apparatus (Bio-Rad Laboratories Inc., Hercules, CA), and probed with either rabbit polyclonal anti-TcpA (1:3000 dilution) or anti-TcpP antibody (1:500 dilution) followed by HRP-conjugated anti-rabbit IgG. Expression of desired proteins was then visualized by using Amersham ECL western blotting detection reagents (GE Healthcare, Buckinghamshire, United Kingdom), according to the product guideline. The signal intensity of specific protein bands were determined with the ImageJ software (http://imagej.nih.gov/ij/), and normalized to that of wild type without anethole samples.

### Rabbit ileal loop assay

*In vivo* effect of anethole on CT production was analyzed by a rabbit ileal loop (RIL) assay using New Zealand white male rabbits (ca 1.8 kg) as described previously [23]. Briefly, overnight fasted rabbits were anesthetized by intramuscular injection of 45 mg/kg ketamine (Ketalar; Daiichi Sankyo Co., Ltd., Tokyo, Japan) and 5 mg/kg xylazine (Selactar; Bayer Healthcare, Leverkusen, Germany). Laparotomy was performed in the anesthetized animals and 8 loops (~8 cm long) with a ~3 cm-inter loop were ligated. Exponential phase growth culture of O1 El Tor variant strain (CRC41) was washed and suspended in PBS (pH 7.0). Ligated segments of RILs were then inoculated with fresh CRC41 culture (10^8^ CFU/loop), either in the presence (0.08–10 mg per loop) or absence of anethole (1% methanol). Loops were then placed back in the peritoneal cavity. After 6 h incubation, the animals were euthanized by injecting 200 mg/kg pentobarbital (Nembutal; Dainippon Sumitomo Pharma Co., Ltd., Osaka, Japan). Fluid accumulation (FA) ratio (ml of fluid per cm of the loop), total bacterial number by plating on thiosulfate-citrate-bile salts-sucrose (TCBS) agar (Eiken Chemical Co., Ltd., Tokyo, Japan) plates and total amount of CT produced in intestinal fluid of each loop by bead-ELISA were then measured. RILs without apparent fluid accumulation were also washed internally with PBS and the washings were analyzed for total bacterial count and CT production. All animal experiments were performed according to the Guidelines for Animal Experimentation of Osaka Prefecture University and approved by the Animal Experiment Committee of Osaka Prefecture University.

### RNA extraction and qRT-PCR

Total RNA was extracted from *V*. *cholerae* cells grown under desired conditions by using TRIzol reagent (Invitrogen), according to the product guidelines. Isolated RNA was treated with RNase-free DNase I (1 U/μg, amplification grade; Invitrogen) to avoid genomic DNA contamination. The reverse transcription was performed from 1 μg of RNA, according to the instruction of quick RNA-cDNA kit (Applied Biosystems Inc.). The obtained cDNA was diluted 1:4 with nuclease-free water, and 4 μl was used for qRT-PCR assay. The qRT-PCR assay was carried out by following the TaqMan probe method with each gene-specific primers, probes and TaqMan Gene Expression master mix (Applied Biosystems Inc.). PCR conditions were 50°C for 2 min, 95°C for 10 min and 40 cycles, each having 95°C for 15 sec and 60°C for 1 min in an ABI PRISM 7500 sequence detection system (Applied Biosystems Inc.). Genomic DNA and DNase-treated RNA that had not been reverse transcribed were used as positive and negative controls, respectively. The housekeeping gene *recA* was used as an internal control. The relative transcription of each gene in comparison with the internal control was analyzed as described previously [24].

### Mutant construction and complementation assay

Construction of *cyaA* and *crp* deletion mutants in the strain CRC41 was performed by following in-frame deletion mutagenesis as described previously [25]. Briefly, deletion in each gene mentioned earlier was generated by an overlapped fusion PCR using the primers listed in Table 2. Then, the *cyaA* and *crp* genes with desired deletion were cloned into the MCS of the suicide vector pWM91 [26] to construct the plasmids pΔ*cyaA* and pΔ*crp*, respectively. The resulting plasmids were then electroporated into *E*. *coli* strain DH5αλpir for maintaining and subsequently to the SM10λpir [27] to mobilize into the *V*. *cholerae* strain CRC41 via conjugation. Resolution of the cointegration was done by sucrose-encounter selection. Recombination and the loss of the wild-type allele were confirmed by PCR using primers flanking the deletions. pCyaA and pCRP were constructed in pBR322 vector to analyze the complementation effect of the proteins in respective *V*. *cholerae* mutants. To construct pCyaA, a 2.65-kb fragment that include entire *cyaA* gene (VC0122) with 126-bp upstream of putative promoter region, was amplified and cloned in to the compatible sites of pBR322. By following the same procedure entire *crp* gene (VC2614) including 183-bp of putative promoter region was cloned in to pBR322 to construct pCRP.

## Results

### Inhibition of CT and TCP production by anethole

**Fig 1:**
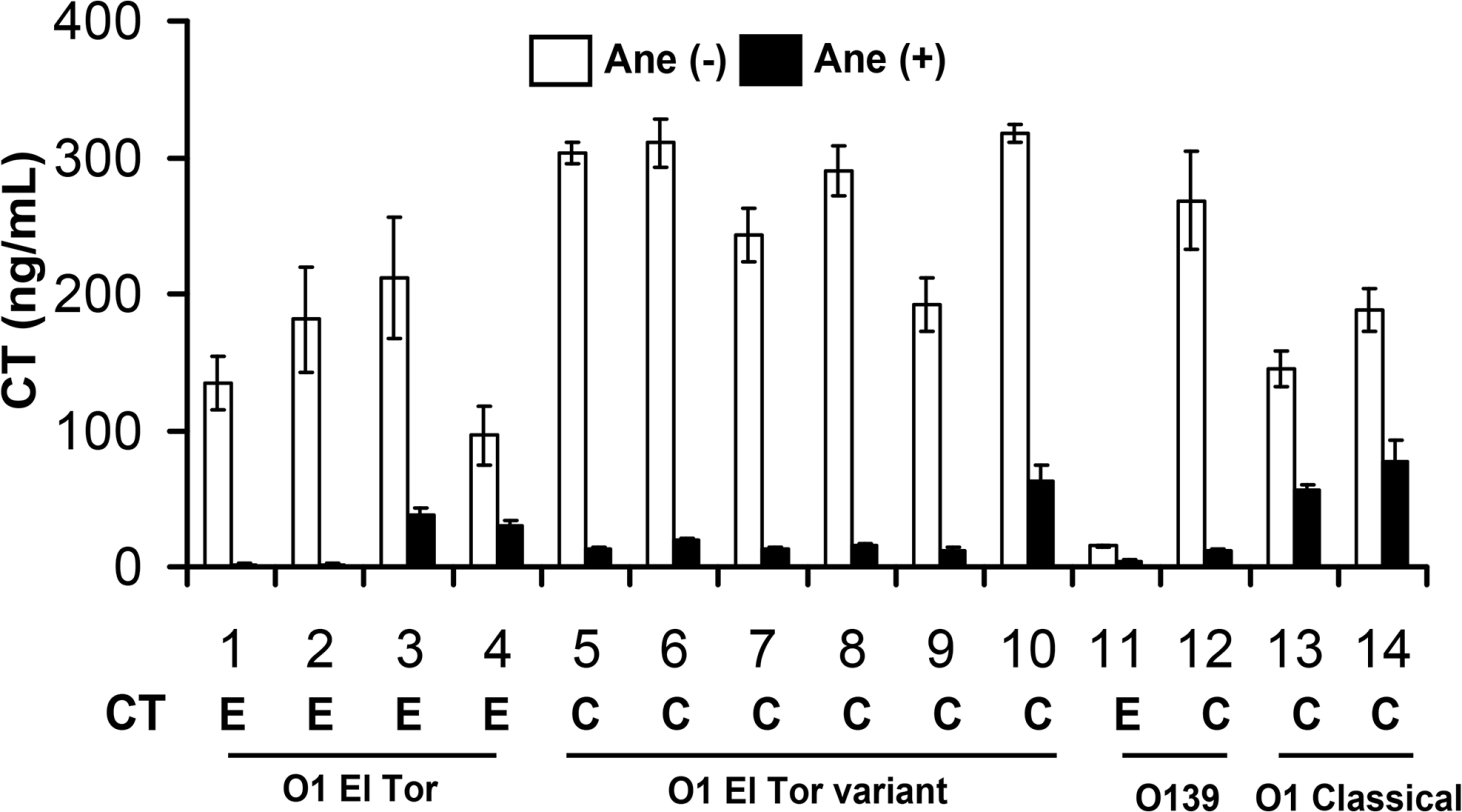
Effect of anethole on CT production in *V*. *cholerae*. Anethole (50 μg/ml) drastically inhibited CT production in various serogroups and biotypes of *V*. *cholerae*. Open and close bars indicate CT production level as ng/ml without and with anethole, respectively. Numerical values in the *x*-axis represent the strain identity (see Table 1). ‘E’ and ‘C’ represent the presence of El Tor and classical type *ctxB* gene allele, respectively in the analyzed strains. Serogroups/biotypes of the *V*. *cholerae* strains are described below the respective *ctxB* allele. Values represent the averages ± SD of three independent experiments.

**Fig 2:**
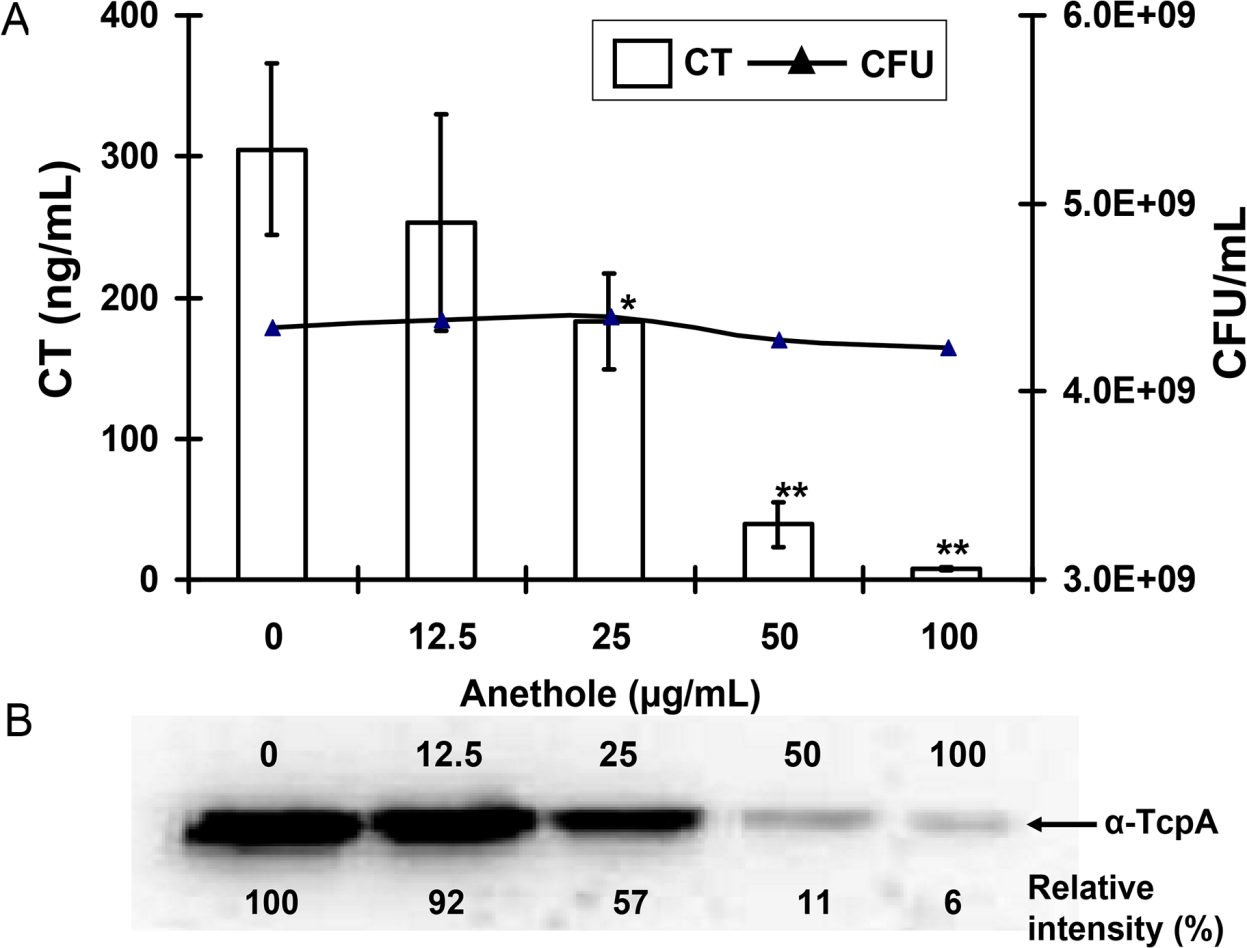
Dose-dependent inhibitory effects of anethole on CT and TCP production in *V*. *cholerae* O1 El Tor variant strain CRC41. (A) Effects of anethole on CT production and bacterial viability presented in the primary and secondary *y-axis*, respectively. *x-axis* indicates the concentrations of anethole used in these assays. Data are presented as the averages ± SD of three independent observations. By using two sample *t*-test, a single asterisk (*) represents *p* <0.05 and two asterisks (**) represents *p* <0.01 as compared with the anethole-free culture. (B) Dose-dependent effect of anethole on TcpA expression. Three independent experiments were conducted and a representative western blot image is shown here. The band signal intensities (shown below the image) of the image of western blot were quantified by ImageJ software (http://imagej.nih.gov/ij/), and normalized to that of without anethole sample (arbitrarily taken as 100%).

We tested the effect of anethole on the production of CT and TCP by culturing strains in presence of sub-bactericidal concentration of anethole. Results showed a significant inhibitory effect of anethole on the production of both CT and TCP (Figs 1 and 2). CT in the culture supernatant fluid was estimated using bead-ELISA, whereas the expression of TcpA was assessed by western blot analysis. Addition of 50 μg/ml anethole inhibited CT production in all strains irrespective of the serogroups or biotypes, although there were apparent variation in the degree of CT production and inhibition by anethole for different strains. To select an optimal concentration of anethole that would not affect the bacterial growth, we tested the effect of anethole on the growth of 14 different *V*. *cholerae* strains and found that up to 100 μg/ml of anethole did not have any detectable effect on the growth of these strains (S1 Table).

Next, dose-dependent effect of anethole on CT production was analyzed in a representative high CT-producing O1 El Tor variant strain CRC41. We found that CT production was inhibited in the presence of anethole (≤100 μg/ml) in a dose-dependent manner. As shown in Fig 2A, 50 and 100 μg/ml anethole could suppress 85% and 95% of CT respectively, compared to anethole-free control culture. Furthermore, these concentrations had no significant effect on CRC41 growth (Fig 2A). Taken together, these results indicated that suppression of CT production in CRC41 by anethole was not due to bacterial growth inhibition. Furthermore, we did not observe any reduction of CT quantity, when we incubated a known concentration of purified CT with either anethole (50 μg/ml) or its solvent methanol (0.5%) at our experimental set up (data not shown). So, we denied a possibility that anethole or its solvent methanol directly acts on CT to cause alteration of its immunological property under our experimental conditions, and also indicated that anethole might affect virulence regulatory cascade to inhibit CT and TcpA expression in *V*. *cholerae*.

Since CT expression is coordinately regulated with the expression of TCP [28] in toxigenic *V*. *cholerae*, expression of TcpA (the major subunit of TCP) was also examined. For this purpose, western blot analysis was carried out from the same CRC41 cultures, used to see the dose-dependent effect of anethole on CT production inhibition. As expected, reduction of TcpA expression was observed (Fig 2B) and well correlated with CT inhibition by anethole (Fig 2A). TcpA expression was reduced ~89% (determined with the ImageJ software; see Methods section) in presence of 50 μg/ml anethole, compared to the anethole-free culture. Thus, anethole inhibited the expression of both of the two major virulence factors of toxigenic *V*. *cholerae*.

### Anethole inhibits CT production irrespective of the culture conditions in AKI medium

**Fig 3:**
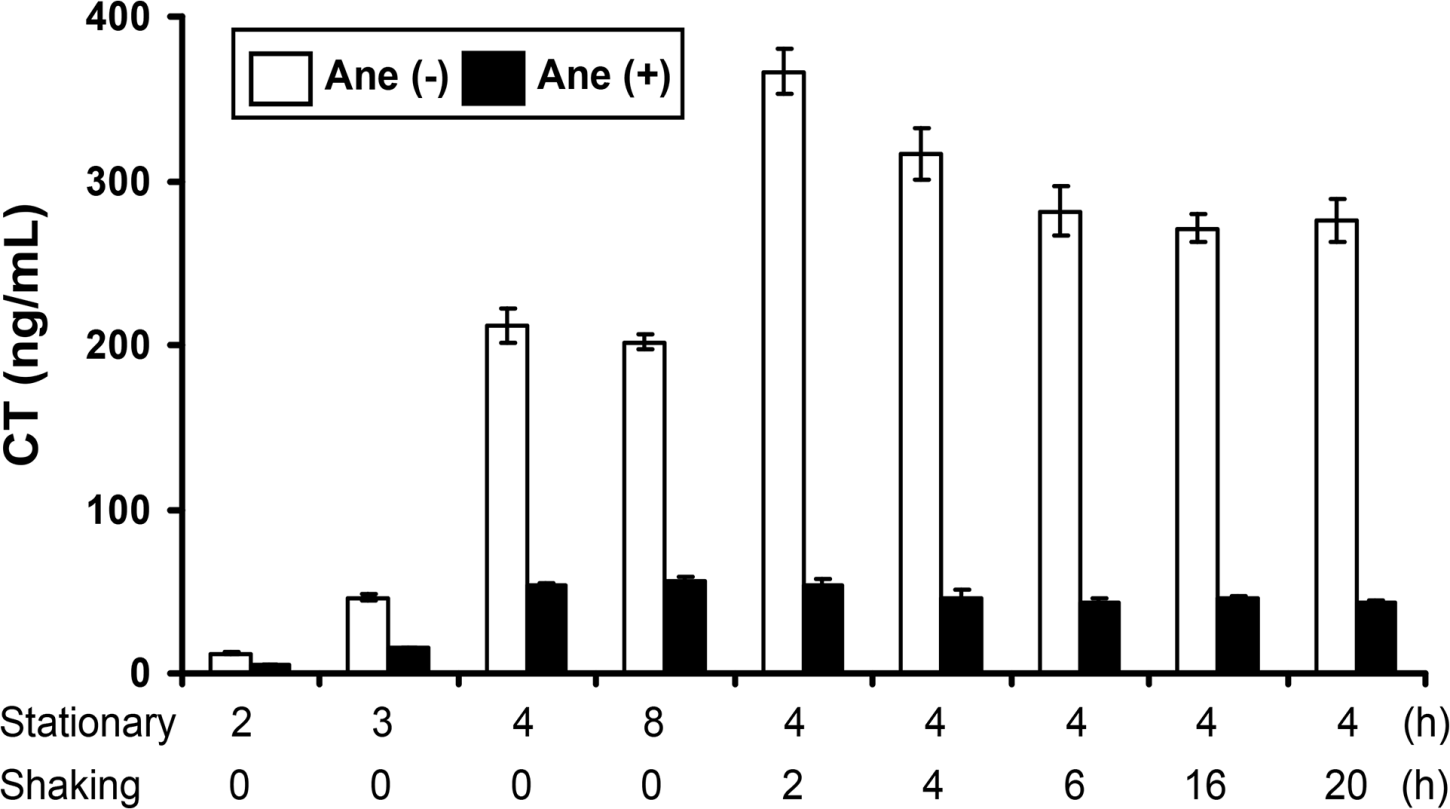
A time-course effect of anethole on CT production in *V*. *cholerae* strain CRC41. CT production was estimated from the CFS of initial stationary and followed by different length of shaking conditions, both in the presence (50 μg/ml) and absence of anethole. Open and close bars indicate CT production level as ng/ml without and with anethole, respectively. Results represented as the mean ± SD of three independent experiments.

Previous studies suggested that initial stationary condition plays a crucial role in the initiation of CT production by El Tor biotype strains in AKI medium supplemented with 0.3% NaHCO_3_, and an enhanced production of CT was noticed following 4 h stationary culture [20, 29]. To analyze the trend of anethole-mediated CT inhibition in the strain CRC41, a time-course assay of CT production with initial stationary and followed by various length of shaking conditions was conducted. We found that CT production in the absence of anethole peaked at 2 h of shaking culture following initial stationary condition, and a high amount of CT was induced at the end of 4 h stationary phase (Fig 3). Further extension of stationary phase (up to 8 h) did not increase the amount of CT production compared to that of 4 h stationary phase. However, addition of anethole inhibited CT production under these conditions.

*V*. *cholerae* cells exposed to very low level of oxygen at stationary growth condition at 37°C in AKI medium supplemented with 0.3% NaHCO_3_ might resemble the environment of host small intestine during the course of infection. In a time-course study, although CT production by the strain CRC41 was found maximum at 2 h of shaking following initial stationary condition (Fig 3), this condition differs from what happen during the course of *V*. *cholerae* infection in natural system. So, CT inhibition regulation studies by anethole at stationary culture in AKI medium supplemented with 0.3% NaHCO_3_ might have significant impact on better understanding of the pathogenic potential of *V*. *cholerae* during early phase of infection in human small intestine.

### Anethole inhibits fluid accumulation caused by toxigenic *V*. *cholerae* in rabbit intestine

**Fig 4:**
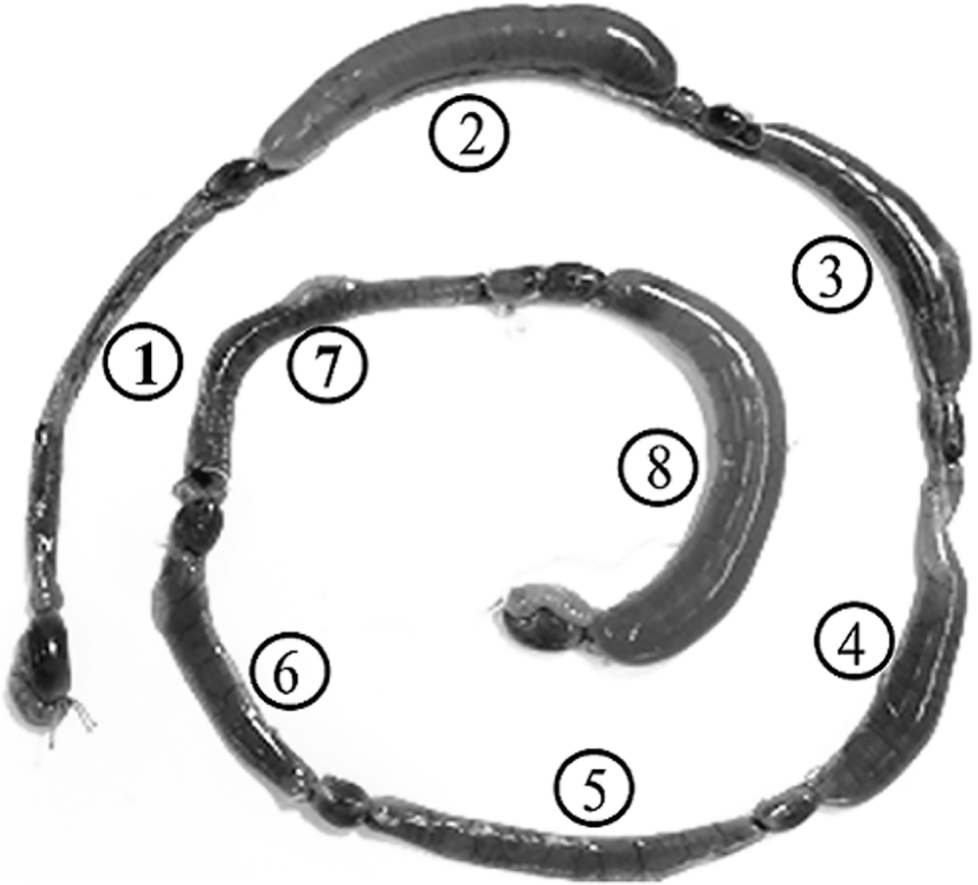
Dose-dependent effects of anethole on toxigenic *V*. *cholerae* (CRC41)-mediated fluid accumulation in ligated rabbit ileal loops (RILs). This RIL image is a representative of three independent observations. Inoculum size, fluid accumulation (F/A) ratio, bacterial colonization and CT production of each loop are summarized in Table 3.

To test whether anethole can effectively suppress expression of CT by toxigenic *V*. *cholerae in vivo*, we used the rabbit ileal loop (RIL) assay, and monitored fluid accumulation, CT production and *V*. *cholerae* viability. As shown in Fig 4 and Table 3, there was marked reduction in fluid accumulation when various sub-lethal doses of anethole were administered together with 10^8^ CFU of a toxigenic strain CRC41 as compared to loop in which bacteria were inoculated without anethole. Thus, CT production as indicated by fluid accumulation was inhibited by anethole in a dose-dependent manner under *in vivo* conditions. There was no significant difference in bacterial counts in the ileal loop fluids, recovered from assays with and without anethole, suggesting that reduced fluid accumulation was not due to bacterial growth inhibition, but for reduced virulence expression in *V*. *cholerae* by anethole.

**Table 3:** Effects of anethole on fluid accumulation, CT production and *V*. *cholerae* (CRC41) viability in rabbit ileal loops.

Loop	Inoculum^a^(CFU* + ane** in mg)	Fluid accumulation ratio^b^(fluid in ml/length in cm)	Recovered bacteria^c^(Total CFU)	Total CT production^d^(ng)
1	PBS	ND	<10	Not det
2	10^8^ + 0.078	0.53 (± 0.09)	1.8 (± 0.17)X10^9^	180.7 (± 6.5)
3	10^8^ + 0.156	0.27 (± 0.03)	1.3 (± 0.05)X10^9^	35.7 (± 7.5)
4	10^8^ + 0.312	0.17 (± 0.02)	2.9 (± 2.00)X10^8^	9.3 (± 0.6)
5	10^8^ + 0.625	0.03 (± 0.03)	1.4 (± 0.07)X10^8^	2.7 (± 0.6)
6	10^8^ + 2.5	ND	1.1 (± 0.12)X10^8^	Not det
7	10^8^ + 10	ND	9.2 (± 2.90)X10^7^	Not det
8	10^8^ + 0^#^	0.66 (± 0.11)	2.6 (± 0.67)X10^9^	330.3 (± 28)

^a^*colony forming unit

**anethole

^#^(1% methanol)

^b^ND, Not determined (significant amount of fluid was not accumulated)

^c^<10, no CFU was detected in 100 μl of PBS washing samples

^d^Not det, Not detected (significant amount of CT was not detected in fluids/washings). In all cases, values presented as the mean with ± SD of three independent rabbit experiments.

### Anethole suppresses the virulence regulatory cascade of *V*. *cholerae* by down regulating TcpP expression at the transcriptional level

To determine at which point of the virulence regulatory cascade anethole affected CT and TCP expression, transcription level of various regulatory genes, including *toxT*, *toxR*, *toxS*, *tcpP*, *tcpH* and *hns* were analyzed via qRT-PCR assay. The relative transcription level of each gene was normalized with that of housekeeping gene *recA*, which was used as an internal control.

Based upon our initial experiments showing the trend of inhibition of CT expression (Fig 3), a culture condition with 4 h stationary which resembles the environment of host small intestine, and followed by 2 h of shaking (at which point CT production by CRC41 was found maximum) were chosen for assaying the expression of the various virulence genes as well as the regulatory genes, in the presence of 50 μg/ml anethole.

**Fig 5:**
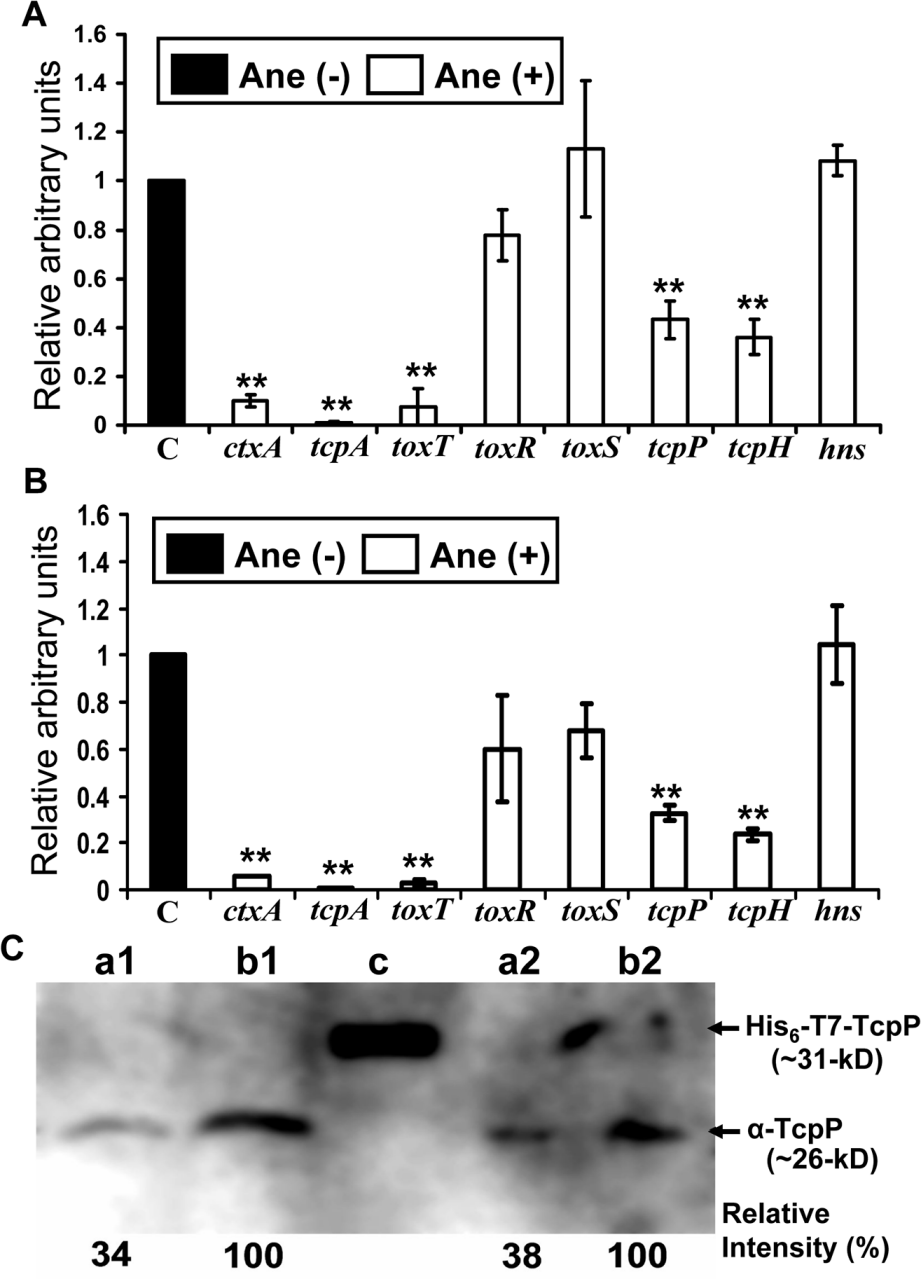
Effect of anethole on the expression of virulence regulatory genes in *V*. *cholerae* strain CRC41. qRT-PCR assay of the genes belonging to virulence regulatory cascade was performed with *V*. *cholerae* cells cultured (A) at 4 h of stationary and (B) followed by 2 h of shaking conditions, both in the presence (50 μg/ml) and absence (0.5% methanol) of anethole. ‘C’ indicates the control value of each target gene transcription, obtained from without anethole sample (arbitrarily taken as 1). Data are presented as the average ± SD of three independent experiments. By using two-sample *t*-test, a double asterisk (**) represents *p* <0.01 as compared with anethole untreated control. (C) Detection of TcpP by western blotting. Lanes ‘a’ and ‘b’ indicate TcpP (~26-kD) expression level in the presence (50 μg/ml) and absence (0.5% methanol) of anethole, respectively. Lane ‘c’ indicates the recombinant His_6_-T7-thrombin digestion site tagged TcpP (~31-kD), which was used as a positive control for detection of TcpP (See Methods section). In left panel (lanes a1 & b1), proteins were obtained from initial 4 h of stationary culture and right panel (lanes a2 & b2), proteins were from initial stationary followed by 2 h of shaking culture. This image is a representative of the three independent observations. The relative band signal intensities (shown below the image) of the image of western blot was quantified by ImageJ software, and normalized to that of without anethole sample (arbitrarily taken as 100%).

As shown in Fig 5A, in presence of anethole at initial stationary culture, *ctxA* gene transcription was repressed ~10-fold. Transcription of major colonization factor *tcpA* was also repressed ~60-fold. At this stage, transcription of other virulence regulatory genes was also repressed by anethole, at various extents: *toxT* (~13-fold), *tcpP* (2.3-fold) and *tcpH* (2.8-fold). On the other hand, transcription of *toxR*, *toxS* and *hns* were not affected significantly. Under shaking condition (Fig 5B), the transcriptions of all the genes analyzed showed the same trend as observed under stationary condition. The transcription of housekeeping gene *recA* was not affected in presence of anethole compared to the untreated controls at either culture conditions (data not shown).

The validity of the qRT-PCR data was further verified by analyzing the expression of TcpP both in the presence and absence of anethole (50 μg/ml). The relative signal intensities of TcpP by western blot (Fig 5C) correlated well with the observation of *tcpP* transcription by qRT-PCR (Fig 5A and 5B). The relative expression of TcpP in the anethole treated cells were 34% (determined with the ImageJ software) and 38% at 4 h of stationary and followed by 2 h of shaking conditions, respectively compared to those of anethole untreated controls.

### Anethole might affect cAMP-CRP signaling system to suppress *tcpPH*

Since there are also upstream regulatory genes for *tcpPH*, the effect of anethole on the transcription of probable upstream regulators was also examined. *tcpPH* are overlapping operons and positively regulated by the membrane-located transcription factor AphA/AphB in *V*. *cholerae* [30]. The cyclic AMP (cAMP)-cAMP receptor protein (CRP) complex has overlapping binding sites with AphA and AphB in *tcpPH* promoter and can negatively regulate the expression of *tcpPH* [31, 32], whereas the quorum sensing regulator HapR has a negative effect on AphA [33]. Therefore, we also analyzed the transcription of possible regulators of *tcpPH*, including *cyaA*, *crp*, *hapR*, *aphA* and *aphB* in the presence of anethole.

**Fig 6:**
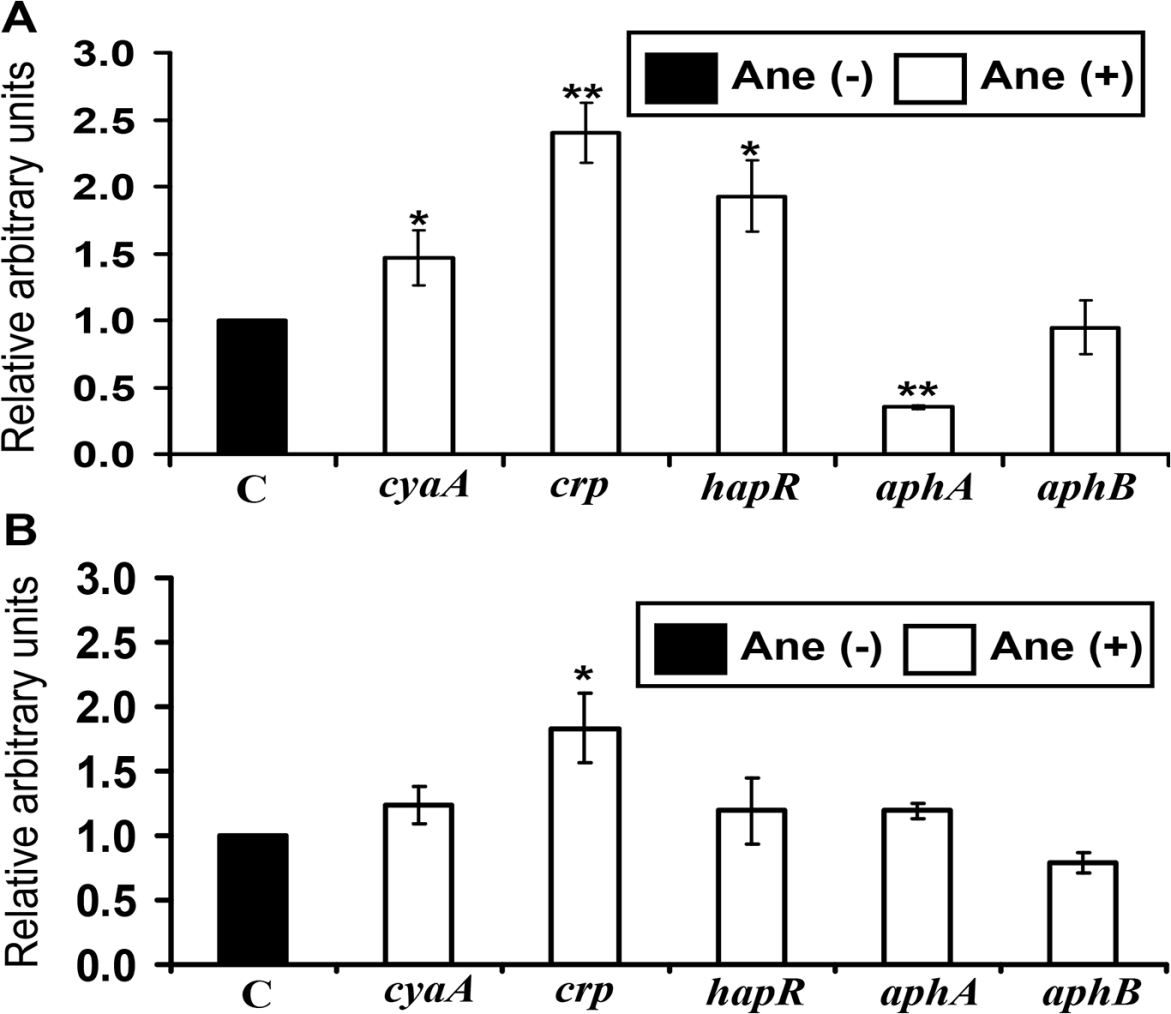
Effect of anethole on the transcription of *tcpPH* regulatory genes in *V*. *cholerae* strain CRC41. Relative transcription level of the *tcpPH* regulatory genes were examined both in the presence (50 μg/ml) and absence (0.5% methanol) of anethole, with *V*. *cholerae* cells cultured (A) at 4 h of stationary and (B) followed by 2 h of shaking conditions. ‘C’ indicates the control value of each target gene transcription, obtained from without anethole sample (arbitrarily taken as 1). Data are presented as the average ± SD of three independent experiments. By using two-sample *t*-test, a single asterisk (*) represents *p* <0.05 and a (**) represents *p* <0.01 as compared with anethole untreated control.

As shown in Fig 6A, after 4 h stationary condition, the relative transcription of *cyaA* (1.5-fold; *p* <0.05), *crp* (2.4-fold; *p* <0.01) and *hapR* (1.8-fold; *p* <0.05) increased but *aphA* (2.8-fold; *p* <0.01) decreased significantly in the presence of 50 μg/ml anethole compared to those of cultures without anethole. Although we observed certain variation in expression of these genes under stationary and shaking condition (Fig 6B), the transcription of *crp* remained consistently elevated in presence of anethole irrespective of the culture conditions. Taken altogether, a hypothesis can be raised that anethole might initiate inhibition of *tcpPH* transcriptions as well as CT through cAMP-CRP complex-mediated signal.

## Discussion

Search for natural compounds with inhibitory effect on bacterial virulence is important particularly in view of growing multidrug resistance among bacterial pathogens. Since ancient times, natural products such as spices, herbs, etc. have been used to treat diarrheal diseases [34]. Moreover, especially in the Indian subcontinent where cholera is endemic from ancient times, people usually take sweet fennel seeds (natural reservoir of anethole) after meal as a gastrointestinal refreshener [35]. The scientific reason behind that is not clearly understood. Although antimicrobial activities of anethole against some bacteria, yeast and fungi have already been reported [19, 36, 37], still there is no report regarding its effect on virulence factors production in *V*. *cholerae*. The present study, demonstrating the inhibitory effect of anethole on virulence expression of *V*. *cholerae* could be an explanation for the traditional use of sweet fennel seeds by the Indian subcontinent people.

It has been already evident that O1 El Tor variant strains are now predominant, and they produce more CT compared to prototype El Tor [7, 38]. Most of the O1 El Tor variant strains tested in this study also showed the same trend in terms of their CT production level (Fig 1). Based upon our previous reports [17] and anethole-mediated *in vitro* CT inhibition data in this study, a high CT-producing O1 El Tor variant strain CRC41 was selected for *in vivo* as well as anethole-mediated CT inhibition regulation studies.

In our preliminary experiments, we observed that 10^8^ CFU of CRC41 could accumulate significant amount of fluid in ligated RIL within 6 h of incubation (S1 Fig). Relatively short incubation period was considered because of the possibility of absorption or degradation of anethole in intestine. As TCP is the major colonization factor and its expression was also repressed by anethole (Fig 2B), some extent of colonization defect of CRC41 in ligated RIL by anethole is expected. But, we observed that although recovered bacteria from the loop inoculated with 0.156 mg of anethole was not drastically varied, total CT production was suppressed ~10-times compared to the anethole-free ‘positive control’ loop for fluid accumulation (Table 3), indicating that anethole inhibited fluid accumulation or CT production in ligated RIL without affecting bacterial viability significantly, as demonstrated by *in vitro* experiments.

It has been reported that at stationary condition in AKI medium, HCO_3_^-^ could stimulate CT production in O1 El Tor strains by enhancing ToxT activity post-translationally [29, 39]. In our present study, inhibition of transcription of *ctxA* along with *tcpA* and *toxT* (Fig 5A), suggested that anethole affects the virulence regulatory cascade prior to *toxT*. These results also ruled out the possibility of interfering anethole with the activity of HCO_3_^-^ in AKI medium, as HCO_3_^-^ enhanced ToxT activity post-translationally. In a previous study, synthetic compound virstatin inhibited CT production by affecting ToxT post transcriptionally [14]. Histone-like nucleoid structuring protein (H-NS) encoded by the gene *hns*, is a basal repressor of many genes including *ctxA*, *tcpA* and *toxT* in *V*. *cholerae* [40]. Previous study demonstrated that in presence of bile, enhancement of H-NS production can repress *ctxA* and *tcpA* transcriptions in a ToxT-independent manner [13]. Recently, we have also reported that capsaicin inhibits *ctxA* and *tcpA* transcriptions by upregulating *hns* transcription, but in a ToxT-dependent fashion [17]. In this study, we did not find any significant enhancement of *hns* transcription in presence of anethole.

Although ToxR plays a very crucial role for the activation of *toxT* in *V*. *cholerae*, only ToxR is not sufficient for the activation of *toxT* [9]. On the other hand, overproduction of TcpP obviates the requirement of ToxR, and can alone activate the *toxT* promoter [11]. In our present study, we observed a drastic repression of *tcpPH* in inhibiting CT, despite any significant changes in *toxR/toxS* transcripts level in presence of anethole (Fig 5A and 5B), also suggesting a *toxR*-independent but *tcpP*-dependent inhibition of *toxT*. Moreover, low level detection of TcpP (Fig 5C) in anethole treated cells compared to the untreated control further confirmed our observations.

Transcriptional analyses of the upstream regulatory genes of *tcpPH* (Fig 6A) suggested that anethole might initiate inhibition of *tcpPH* transcriptions as well as CT by affecting quorum sensing regulatory genes via cAMP-CRP complex-mediated signal at stationary phase. Apart from generating quorum sensing signal, cAMP-CRP complex could directly inhibit *tcpPH* promoter activity by binding to the competitive site for AphA and AphB [32]. However, at shaking stage (Fig 6B) although significant elevation of *crp* was observed, transcription of other *tcpPH* regulatory genes were not affected by anethole. This suggested that cAMP-CRP complex mediated activation of quorum sensing pathway is terminated at this stage. It has been reported that although initial expression of ToxT depends on the activity of ToxR and TcpP, once produced, ToxT itself is able to maintain its expression by activating distal *tcpA* promoter (auto-regulatory loop) leading to transcription of *toxT* [41, 42]. Taken together, it can be hypothesized that in anethole exposed cells, activation of cAMP-CRP signaling system leads to a very low level production of TcpP, which might fail to activate *toxT* transcription initially and subsequently prevent the activation of auto-regulatory loop of *toxT* transcription. Thus, due to the failure of activation of auto-regulatory loop of *toxT* transcription at initial stationary phase, further activation of *toxT* transcription (Fig 5B) might not occur in anethole treated cells, and thus contribute to suppress CT production in *V*. *cholerae* at aerobic growth phase.

To further investigate the role of cAMP-CRP signaling system in anethole-mediated suppression of CT expression, we constructed Δ*cyaA* and Δ*crp* isogenic mutants of the strain CRC41. These mutants were apparently slow growing with increased doubling times in AKI medium at 37°C as well as showed a phenotypic growth defect on TCBS agar compared to the wild-type strain. But, complementation of the mutations by transformation with recombinant plasmids carrying the cloned *cyaA* and *crp* genes (pCyaA and pCRP, respectively) restored their growth rate to the level of the wild type strain (data not shown). Unfortunately, we failed to analyze the effect of anethole on CT production in these mutants, since they showed significant growth inhibition at 50 μg/ml anethole compared to anethole-free culture (S2 Fig).

Here, we showed evidences that anethole might initiate CT production inhibition in *V*. *cholerae* by activating cAMP-CRP signaling system. However, along with cAMP-CRP complex mediated signal, contribution of other factors in anethole-mediated virulence suppression could not be excluded. Recently, it has been reported that extracytoplasmic stress response can induce integral membrane zinc metalloprotease RseP (formerly known as YaeL protease) in *V*. *cholerae*, which causes degradation of TcpP [22, 43]. It is possible that anethole could induce extracytoplasmic stress response and thereby induce the expression of some proteases, such as RseP or major serine proteases DegS in *V*. *cholerae*. But, we failed to find any significant differences of *rseP* and *degS* in their transcription level between anethole-treated and untreated cells (S3 Fig). Due to anethole-mediated *tcpH* suppression proteolytic cleavage of TcpP could be another possibility, as TcpH protects the periplasmic domain of TcpP from proteolytic cleavage [12]. It has also shown that at non-permissive conditions, a protein named PepA partially inhibits *tcpP* transcription in *V*. *cholerae* [44]. *tcpP* promoter can also be negatively regulated by PhoB, which binds to a distinct site from both the AphA and AphB binding sites [45]. Moreover, oxidative modification of AphB could repress virulence expression in *V*. *cholerae* by affecting *tcpP* transcription [46]. So, involvement of synergistic activation of any of the mentioned pathways along with the cAMP-CRP signaling system in anethole-mediated virulence suppression in *V*. *cholerae* could not be excluded, unless tested.

**Fig 7:**
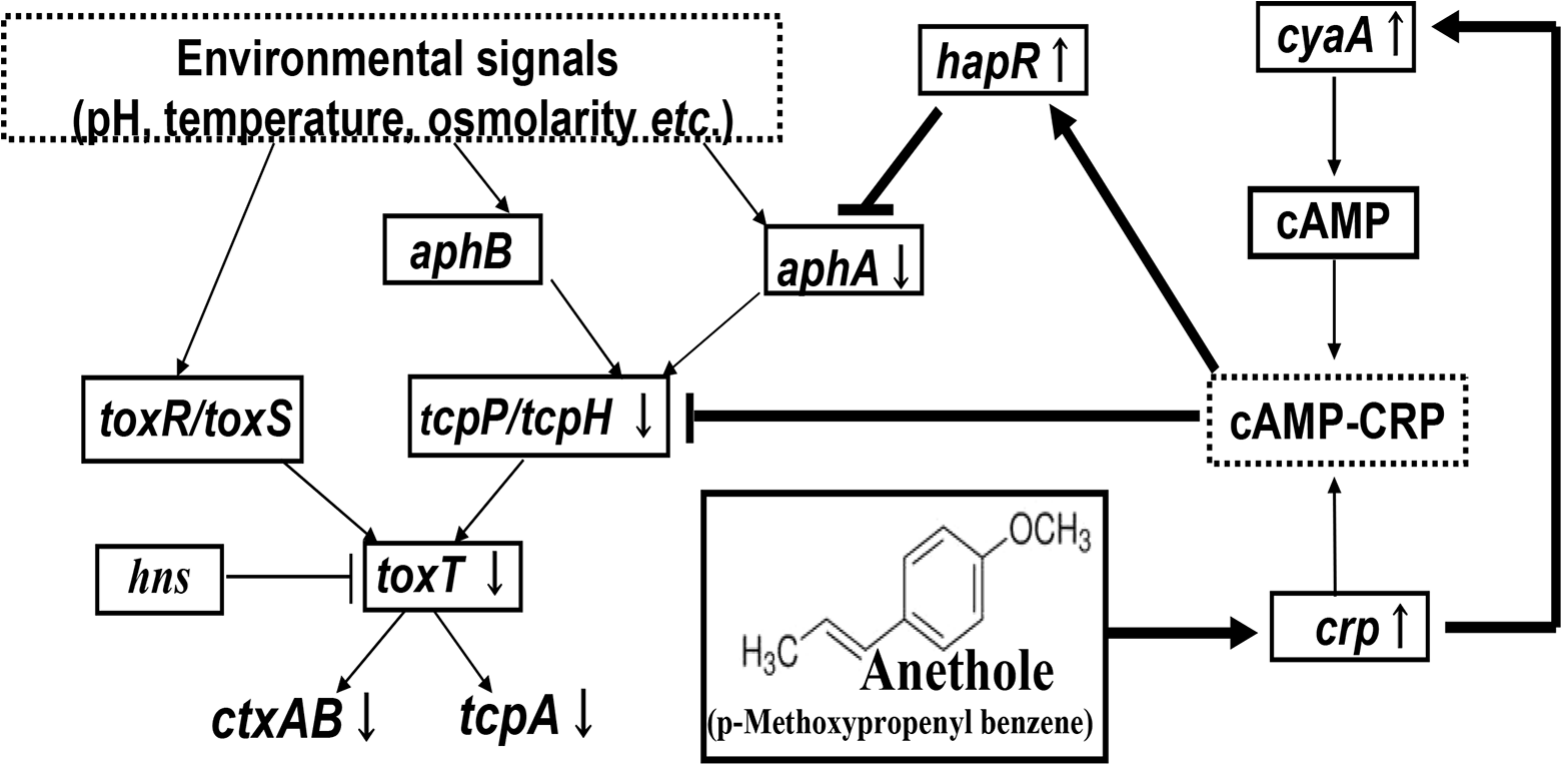
The hypothetical regulatory cascade of CT production inhibition in *V*. *cholerae* by anethole. In all cases, arrows indicate positive regulation while bars denote negative or inhibitory effects. Arrows besides the genes name represent significant increase and decrease of transcription of specific gene, in presence of anethole. Thick arrows represent the anethole-mediated effect on *tcpPH* suppression.

Based upon the observations in this study, we propose a model showing the hypothetical mechanisms of how anethole-mediated signal affect the general *toxR* regulon of CT expression (Fig 7). In this scenario, anethole might initiate CT production inhibition by activating *crp* at stationary phase grown *V*. *cholerae* cells. In response to *crp*, transient activation of *cyaA* might occur. Then, cAMP-CRP complex could exert dual inhibitory effect on *tcpPH* promoter either binding directly or by activating quorum-sensing regulatory genes. Thus, very low level expression of TcpP inhibits initiation of *toxT* transcription directly and subsequently prevents the activation of virulence factors production mechanisms in *V*. *cholerae* at later growth phase.

In summary, we have demonstrated that sub-bactericidal concentration of anethole is a potent inhibitor of virulence factors production in *V*. *cholerae*, irrespective of their serogroups and biotypes. CT-mediated fluid accumulation in ligated rabbit intestine was also inhibited by anethole, when co-cultured with recently emerged *V*. *cholerae* O1 El Tor variant strain. Moreover, we have revealed an interesting mechanism of anethole-mediated *in vitro* virulence suppression in *V*. *cholerae*. Anethole drastically inhibited CT and TCP expression by suppressing TcpP at its transcriptional level, remaining *toxR* being unaffected. These findings also supported the previous observation that TcpP is more directly responsible than ToxR for the transcriptional activation of *toxT* [10, 11]. cAMP-CRP complex plays as a global regulator of gene expression in enteric bacteria [47] which includes suppression of CT and TCP expression in *V*. *cholerae* [31]. Based on the results of this study, we have raised a hypothesis that anethole might suppress TcpP and thereby virulence expression in *V*. *cholerae* by activating cAMP-CRP complex mediated signal, might have an impact to design new antibacterial compounds against multidrug resistant bacterial pathogens including *V*. *cholerae*. Although further studies are needed, our data suggest that anethole could be an antivirulence drug candidate against multiple antibiotic resistant toxigenic *V*. *cholerae*-mediated infections.

## Supporting Information

S1 Fig Dose-dependent effects of *V*. *cholerae* O1 El Tor variant strain CRC41 on fluid accumulation in RILs in the presence or absence of anethole. Fresh CRC41 cultures were inoculated and incubated for 6 h in ligated RIL. Loops no. 3, 5 and 7 represent the effect of 10 mg of anethole on fluid accumulation by 10^8^, 10^7^ and 10^6^ CFU of CRC41, respectively.
(TIF)

S2 Fig Effects of anethole (50 μg/ml) on the growth of Δ*crp* and Δ*cyaA* mutants of *V*. *cholerae* O1 El Tor variant strain CRC41. *x-axis* indicates the culture conditions used to analyze the samples. Primary *y-axis* indicates the OD value of the culture at 600nm and secondary *y-axis* indicates the bacterial viability as CFU/ml at desired time points.
(TIF)

S3 Fig Effect of anethole on the transcriptions of zinc metalloprotease encoding *rseP* and major serine protease encoding *degS* in *V*. *cholerae* O1 El Tor variant strain CRC41. Relative transcription level of the genes were examined both in the presence (50 μg/ml) and absence (0.5% methanol) of anethole, with *V*. *cholerae* cells cultured **(A)** at 4 h of stationary and **(B)** followed by 2 h of shaking conditions. ‘C’ indicates the control value of each target gene transcription without anethole (arbitrarily taken as 1). Data are presented as the average ± SD of three independent experiments.
(TIF)

S1 Table Effect of anethole on the growth of different toxigenic *V*. *cholerae* strains. OD_600_, Optical density at 600nm. *V*. *cholerae* cells were co-cultured with different concentrations of anethole in AKI medium with initial 4 h of stationary followed by 4 h of shaking, and OD_600_ of each culture was measured by a spectrophotometer. In all cases, values represent the mean (OD_600_) ± SD of three independent bacterial cultures at respective anethole concentration.
(PDF)
