# Supplementary figures and images for: Suppression of Virulence of Toxigenic Vibrio cholerae by Anethole through the Cyclic AMP (cAMP)-cAMP Receptor Protein Signaling System
Source: PLoS One. 2015 Sep 11;10(9):e0137529. doi: 10.1371/journal.pone.0137529 (PMC4567338; doi:10.1371/journal.pone.0137529)

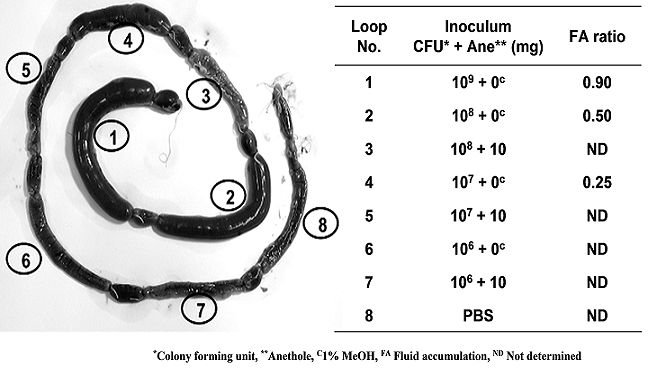

Supplement: S1 Fig — Fresh CRC41 cultures were inoculated and incubated for 6 h in ligated RIL. Loops no. 3, 5 and 7 represent the effect of 10 mg of anethole on fluid accumulation by 108, 107 and 106 CFU of CRC41, respectively. (TIF) [file pone.0137529.s001.tif]

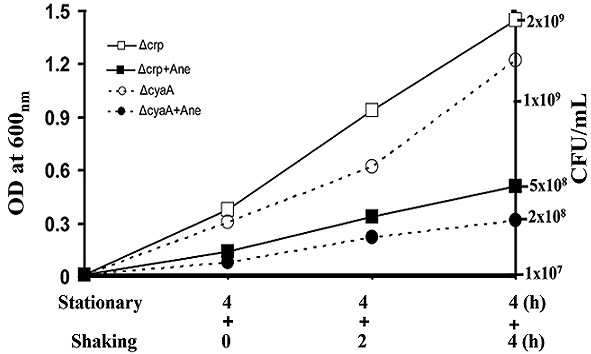

Supplement: S2 Fig — x-axis indicates the culture conditions used to analyze the samples. Primary y-axis indicates the OD value of the culture at 600nm and secondary y-axis indicates the bacterial viability as CFU/ml at desired time points. (TIF) [file pone.0137529.s002.tif]

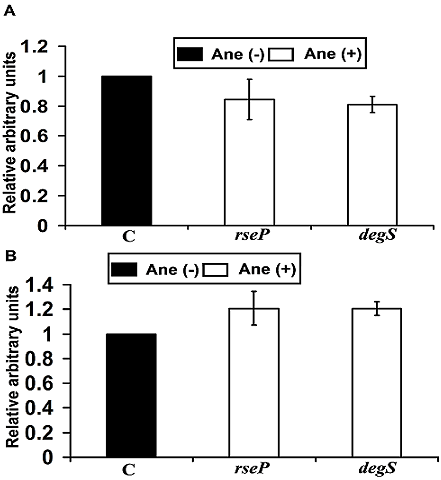

Supplement: S3 Fig — Relative transcription level of the genes were examined both in the presence (50 μg/ml) and absence (0.5% methanol) of anethole, with V. cholerae cells cultured (A) at 4 h of stationary and (B) followed by 2 h of shaking conditions. ‘C’ indicates the control value of each target gene transcription without anethole (arbitrarily taken as 1). Data are presented as the average ± SD of three independent experiments. (TIF) [file pone.0137529.s003.tif]
