# Supplementary material for: Suppression of Virulence of Toxigenic Vibrio cholerae by Anethole through the Cyclic AMP (cAMP)-cAMP Receptor Protein Signaling System
Source: PLoS One. 2015 Sep 11;10(9):e0137529. doi: 10.1371/journal.pone.0137529 (PMC4567338; doi:10.1371/journal.pone.0137529)
Supplement: S1 Table — OD600, Optical density at 600nm. V. cholerae cells were co-cultured with different concentrations of anethole in AKI medium with initial 4 h of stationary followed by 4 h of shaking, and OD600 of each culture was measured by a spectrophotometer. In all cases, values represent the mean (OD600) ± SD of three independent bacterial cultures at respective anethole concentration. (PDF) [file pone.0137529.s004.pdf]

Table S1. Effect of anethole on the growth of different toxigenic *V. cholerae* strains.

Strain ID	OD_{600} at different concentrations of anethole (µg/ml)				
	**0**	**50**	**100**	**150**	**200**
NICED-10	1.80±0.09	1.74±0.05	1.57±0.09	0.67±0.14	0
NICED-3	1.38±0.06	1.37±0.06	1.30±0.07	0.11±0.02	0
P130	1.86±0.11	1.76±0.07	1.68±0.05	0.48±0.09	0
VC190	2.15±0.05	1.94±0.06	1.80±0.10	0.38±0.05	0
AI-091	2.47±0.08	2.40±0.09	2.12±0.11	0.47±0.14	0
CO533	2.60±0.05	2.50±0.10	2.14±0.06	0.52±0.06	0
CRC27	2.46±0.06	2.29±0.06	2.18±0.06	0.61±0.07	0
CRC41	2.59±0.08	2.47±0.07	2.32±0.11	0.75±0.12	0
CRC87	2.12±0.06	1.97±0.07	1.70±0.08	0.14±0.07	0
B33	2.08±0.06	1.87±0.10	1.84±0.07	0.62±0.06	0
SG24	1.90±0.08	1.65±0.08	1.39±0.06	0.37±0.13	0
CRC142	2.39±0.08	2.31±0.10	2.10±0.17	0.70±0.13	0
569B	1.29±0.08	1.22±0.06	1.04±0.07	0	0
O395	1.24±0.06	1.06±0.05	0.80±0.02	0	0

OD_{600}, Optical density at 600nm. *V. cholerae* cells were co-cultured with different concentrations of anethole in AKI medium with initial 4 h of stationary followed by 4 h of shaking, and OD_{600} of each culture was measured by a spectrophotometer. In all cases, values represent the mean (OD_{600}) ± SD of three independent bacterial cultures at respective anethole concentration.
